# The association of serum vitamin D3 and vitamin D binding protein levels before and after treatment with the response to neoadjuvant chemotherapy in Egyptian breast cancer patients: a prospective observational study

**DOI:** 10.1007/s00210-025-04644-4

**Published:** 2025-12-20

**Authors:** Dina Y. El-Shabrawy, Ahmed Hassan, Amal Halim, Laila A. Eissa

**Affiliations:** 1 Department of Biochemistry, Faculty of Pharmacy, Mansoura University, Mansoura, 35516 Egypt; 2 Department of Surgical Oncology Center, Mansoura University, Mansoura, 35516 Egypt; 3 Clinical Oncology and Nuclear Medicine Department, Faculty of Medicine, Mansoura University, Mansoura, 35516 Egypt

**Keywords:** Vitamin D3, Breast cancer, Vitamin D-binding protein, Neoadjuvant chemotherapy, Prognostic biomarkers

## Abstract

**Background:**

Vitamin D regulates cell growth and differentiation, encourages pro-apoptotic effect, stimulates antiangiogenic effect, and affects both innate and adaptive immunity. However, serum vitamin D level could differ between countries according to geographic, genetic, and dietary factors.

**Aim of the work:**

Cosidering that most studies of relations between vitamin D level and breast cancer were conducted outside Africa and considering the fact that populations differ in sun exposure, dietary habits, and genetic construction, this study aimed to investigate the relationship between serum levels of vitamin D3 and vitamin D- binding protein (VDBP) with pathological response, clinicopathological characteristics, and various biological markers in Egyptian breast cancer patients undergoing neoadjuvant chemotherapy (NACT).

**Methods:**

A total of 71 female breast cancer patients (mean age: 57 years) were enrolled in this prospective observational study. Fasting blood samples were collected 1 day before and 3 months after initiation of NACT. Serum levels of vitamin D3 and V DBP were quantified using high-performance liquid chromatography (HPLC) and enzyme-linked immunosorbent assay (ELISA), respectively. Tumor expression of Ki-67, HER2, progesterone receptor (PR), and estrogen receptor (ER) was assessed via immunohistochemistry. Serum levels of CA 15-3 and Bcl-2 were also measured.

**Results:**

Complete pathological response (pCR) was achieved in 44 patients (68.7%). A statistically significant increase in both vitamin D3 and VDBP levels was observed following NACT (*p* < 0.001). Lower pre- and post-treatment levels of vitamin D3 and VDBP were significantly associated with postmenopausal status, higher tumor grade and stage, triple-negative breast cancer subtype, and high Ki-67 expression (*p* < 0.001). Conversely, higher levels were significantly associated with achieving pCR (*p* < 0.001). Both vitamin D3 and VDBP levels demonstrated a significant negative correlation with tumor stage and grade (*p* < 0.001). Among different clinical and laboratory parameters, only triple-negative subtype and baseline vitamin D were significantly predictive for pCR by multivariable analysis (OR 1.488 and 0.506, respectively) and (95% CI 1.109–1.825 and 0.331–0.75, respectively).

**Conclusion:**

Serum levels of vitamin D3 and VDBP significantly increased after NACT and were associated with favorable clinicopathological features and pCR. Only triple-negative subtype and baseline vitamin D were significantly predictive for pCR by multivariable analysis. These findings suggest that vitamin D3 and VDBP may serve as potential prognostic indicators in breast cancer management.

## Introduction

Vitamin D, a fat-soluble steroid hormone, has emerged as a critical factor in cancer biology beyond its well-established role in calcium and phosphate homeostasis. Its active form, 1α,25-dihydroxyvitamin D3, exerts biological effects primarily through binding to the vitamin D receptor (VDR), a nuclear receptor expressed in various tissues, including breast epithelium. Upon activation, the VDR modulates the expression of genes involved in cell proliferation, differentiation, apoptosis, immune regulation, and angiogenesis—all of which are key processes in cancer development and progression. In vitro studies have shown that 1α,25(OH)₂D₃ and its analogues inhibit proliferation and induce apoptosis, often through cell cycle arrest at the G0/G1 phase and modulation of apoptotic regulators such as BAX and BCL-2. Vitamin D also interferes with tumor-supporting pathways by downregulating angiogenic and inflammatory mediators such as VEGF, endothelin-1, Glut-1, and IL-1β (Yoshida and Stern 2012; Moukayed and Grant 2013). 

Epidemiological and clinical studies have reported inverse associations between serum vitamin D levels and breast cancer (BC) risk, disease progression, and treatment resistance. Additionally, genetic polymorphisms in the VDR and vitamin D-binding protein (VDBP)—the main carrier of vitamin D in circulation—have been linked to variations in breast cancer susceptibility and outcomes (Keum et al. 2019; Thomas and Andersen 2023; Tagliabue et al. 2015; Gabriella et al. 2014; Iqbal and Khan 2017; Speeckaert et al. 2006; Rozmus et al. 2020).

Given the growing interest in the interplay between vitamin D status and breast cancer therapy, particularly in the neoadjuvant setting (Abbas et al. 2008; Chiba et al. 2018; Viala et al. 2018; Ottaiano et al. 2024; Lappe et al. 2017), evaluating serum vitamin D3 and VDBP levels may provide valuable insights into treatment response and prognosis.

Considering that most studies of relations between vitamin D level and breast cancer were conducted outside Africa and considering the fact that populations differ in sun exposure, dietary habits, and genetic construction, this study aimed to investigate the relationship between serum levels of vitamin D3 and VDBP with pathological response, clinicopathological characteristics, and various biological markers in Egyptian breast cancer patients undergoing neoadjuvant chemotherapy (NACT).

## Materials and methods

### Study design and population

This prospective observational study included 71 female patients diagnosed with stage II–III BC, treated with neoadjuvant chemotherapy (NACT) at the Oncology Center of Mansoura University between January 2018 and January 2019. The study is approved by the Institutional Research and Ethics Committee (approval code no. 2023-89), and written informed consent was obtained from all participants.

- **Inclusion criteria were** female patients aged 18 to 70 years, histologically confirmed breast cancer (stages II–III), eligible for NACT as per institutional protocol.
- **Exclusion criteria were** intake of vitamin D supplements, history of other malignancies, severe comorbidities, and pregnancy or lactation.
- **Primary outcome:** Association between V D3 levels and treatment response to NACT.
- **Secondary outcomes:** Changes in Bcl-2, CA 15-3, vitamin D3, and VDBP serum levels pre- and post-NACT. Moreover, association and correlation of VD3 levels and different patient, tumor, and laboratory characteristics.

We estimated a sample size of at least 60 patients to detect a moderate correlation (*r* = 0.35) between vitamin D3 levels and response rate with 80% power and *α* = 0.05 (using G*Power software). Our final *n* = 71 provided sufficient power.

### Treatment protocol and response assessment

Stage II and III patients received four cycles of anthracycline-based chemotherapy (doxorubicin 60 mg/m^2^ IV day1 and cyclophosphamide 600 mg/m^2^ day1 cycled every 21 days), followed by four cycles of taxane-based chemotherapy (paclitaxel 80 mg/m^2^ IV cycled every 7 days for 12 weeks. On the other hand, stage I and II were treated by upfront surgery.

Response evaluation in cases who received NACT:

Treatment response was evaluated by the following criteria:

- *Radiological criteria* two weeks after completing NACT by mammography (and MRI in the completely responsive patients) based on RECIST 1.1:
  - **Complete Response (CR):** Disappearance of all target lesions.
  - **Partial Response (PR):** At least a 30% decrease in the sum of diameters of target lesions.
  - **Stable disease**: Neither PR, nor progression
  - **Progressive disease** (PD):>or =20% increase in sum of diameters.

***Pathological criteria***:

Pathological criteria were assessed postoperatively using standard histopathological criteria, including:

- **Pathological complete response** (pCR): No residual invasive carcinoma in breast or lymph nodes.
- **Partial response (residual disease):** Presence of invasive carcinoma in resected tissue.
- **No response**

### Clinical staging and sample collection

Clinical staging was performed preoperatively through physical examination and mammography, with core needle biopsy and metastatic workup through CT chest, abdomen, and pelvis and bone scintigraphy.

Fasting blood samples were collected as a baseline 1 day before initiation of NACT and 3 months after completing NACT. Samples were centrifuged at 2000–3000 rpm for 20 min to obtain serum, which was stored at −70 °C until analysis.

### Laboratory methods

#### Reagents and chemicals

High-performance liquid chromatography (HPLC)-grade methanol, acetonitrile, and orthophosphoric acid were purchased from Sigma-Aldrich (Germany) and Merck (Germany). All reagents used were of analytical grade.

#### Vitamin D₃ assay (HPLC method)

Serum vitamin D₃ levels were determined using HPLC PerkinElmer™ Series 200 chromatograph with UV detection at 250 nm. The column was a reversed-phase C18 column. The mobile phase consisted of acetonitrile/water (99:1, v/v) at a flow rate of 1 mL/min. Sample preparation for human serum was applied through the transfer of 1 mL serum spiked with 0.2 mL of vitamin D₃ (40 µg), and then add 0.5 mL trichloroacetic acid and 0.3 mL acetonitrile. Centrifuge at 4000 rpm for 5 min at room temperature. Twenty microliters of the supernatant was injected into the HPLC system.

#### Serum VDBP

Measured using a commercial ELISA kit (BT Lab, Shanghai, China, catalog no. E1402Hu) per the manufacturer’s protocol.

#### Serum Bcl-2

Serum levels of Bcl-2 were determined using ELISA (catalog no. NBP1-91188). Assays were conducted according to the manufacturer’s instructions.

#### CA 15-3 measurement

Serum CA 15-3 concentrations were determined using an immunoradiometric assay (ELSA-CA 15-3, catalog no. KA0206). Reference values were established using data from patients with benign breast disease.

#### Immunohistochemical assessment of Ki-67, ER, PR, and HER2

For Ki-67, expression was quantified as the labeling index (LI), i.e., the percentage of tumor cells showing positive nuclear staining. A cut-off of 25% was used to distinguish high vs. low proliferation. Tumor sections were analyzed by pathologists using manual counting and image analysis.

### Statistical analysis

Statistical analyses were performed using IBM® SPSS® Statistics version 25 (IBM® Corp., Armonk, NY, USA). All continuous variables were checked for normality using the Shapiro–Wilk test and were found to follow a normal distribution. Numerical data are presented as mean ± standard deviation (SD), and categorical variables are presented as frequencies and percentages. For comparisons between two independent groups, the independent-samples *t*-test was used. For paired data (before vs. after treatment in the same group), the paired-samples *t*-test was used. For comparisons among more than two independent groups, one-way ANOVA was used, followed by Tukey’s post hoc test with Bonferroni correction for multiple pairwise comparisons. Bonferroni correction was applied in post hoc analyses to control for type I error.

Pearson’s correlation coefficient (*r*) was used for correlations between two continuous variables (e.g., vitamin D3 vs. VDBP, vitamin D3 vs. BMI, vitamin D3 vs. age). Spearman’s rank correlation coefficient (*ρ*) was used when one variable was ordinal (e.g., tumor stage, tumor grade) and the other continuous.

Multivariable logistic regression was used to identify independent predictors of complete pathological response (pCR) to NACT. Potential confounders entered into the model included age, menopausal status, BMI, tumor stage, triple-negative status, baseline serum vitamin D3, and baseline VDBP levels. Results are reported as odds ratios (OR) with 95% confidence intervals (CI) and *p*-values. All statistical tests were two-tailed, and a *p*-value < 0.05 was considered statistically significant.

## Results

### HPLC method for total serum concentration of vitamin D3 was established

**Fig. 1:**
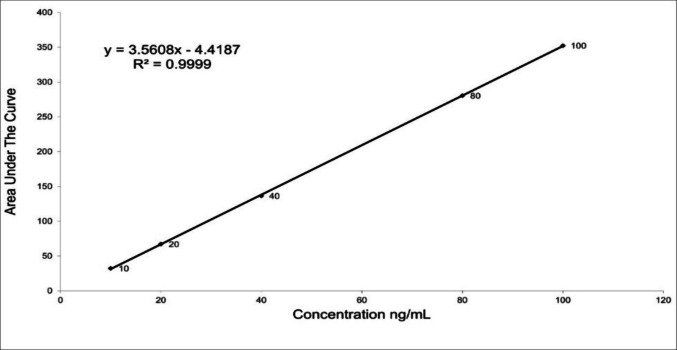
curve for HPLC determination of vitamin D_3_

The method proved to be accurate and precise through evaluating percentage recoveries and relative standard deviations of triplicate determinations; it was 98.67 ± 1.57, respectively, which is not greater than 2 (Higashi et al. 2010). The linearity range in human serum is from 10 to 100 ng/mL, with a correlation coefficient of 0.9999, as shown in the constructed calibration curve (Fig. 1) or regression equation at the same condition.

*Y* = *a* + *b* *C*

*Y* = −4.4187 + 3.5608 *C*

while *Y* is the peak area, *a* is the intercept, *b* is the slope, and *C* is the concentration of vitamin D3 (ng/Ml).

### Demographic, clinical, and laboratory characteristics of subjects (Table 1)

The present study included 71 BC female patients before and after NACT. The mean age was 56.9 years. Stage III was the commonest stage (45%), while infiltrating duct carcinoma was the most common pathological type (83.1%). ER, PR, and HER2 positivity existed in 78.9%, 69%, and 45.1% of cases, respectively, while 14% were triple negative. Ki67 below 20% existed in 52.1% of cases.

The response according to RECIST 1.1 is shown in Table 1. Most cases achieved complete response (CR) (68.7%). The pathologic response rates were similar to the radiologic rates.

**Table 1:** Demographic, clinical and laboratory characteristics

Parameter	No	%	Mean	SD	Median	Range
Age			56.9	+/-12.9		
BMI			36.1	+/-5.6		
Normal weight	3	4.2				
Overweight	5	7				
Obese	36	88.7				
Premenopausal	22	31				
	49	69				
DM	15	21				
Hypertension	28	39.4				
CLD	4	5.6				
Heart disease	8	11.3				
Left sided disease	28	39.4				
Right sided disease	43	60.6				
T stage:						
Tis	3	4.2				
T1	10	14.1				
T2	43	60.6				
T3	14	19.7				
T4	1	1.4				
*N* stage						
N0	22	31.0				
N1	24	33.8				
N2	18	25.4				
N3	7	9.9				
Staging						
CIs	2	2.8				
I	8	11.3				
II	29	40.8				
III	32	45.1				
Pathological Types						
IDC	59	83.1				
ILC	5	7				
DCIS	3	4.2				
Mixed	1	1.4				
Others	4	5.6				
Grades						
Grade1	11	15.5				
Grade 2	48	67.6				
Grade3	12	16.9				
CA15.3					20IU/ml	6–163
Positive ER	56	78.9				
Positive PR	49	69				
Positive HER2	32	45				
Absent triple negative	61	85.9				
High Ki67	34	47.9				
Bcl-2	0	0				
Radiologic response						
Complete response	44	68.7				
Partial response	16	25				
No response /progression	4	6.25				

### The comparison of vitamin D3 and VDBP before and after (NACT therapy with consideration of nutritional and menopausal status

Vitamin D3 and VDBP serum levels increased significantly after NACT therapy (*p* < 0.001 for each) as shown in Table 2. On studying the association of vitamin D3 levels with the menopausal status, it was revealed that postmenopausal women had a significant association with lower vitamin D3 levels before and after treatment (*p* = 0.011 and 0.009, respectively) unlike premenopausal women. The nutritional status was not associated with vitamin D3 neither before nor after treatment. Similarly, postmenopausal patients had significantly lower serum VDBP levels both before and after treatment (*p* = 0.029 and 0.049, respectively) when compared to premenopausal patients. Nutritional status was not associated with VDBP neither before nor after treatment.

**Table 2:** General comparison of vitamin D_3_ and VDBP serum levels before and after treatment and in different nutritional and menopausal states

		BC *N*=71										
		Before						After				
		mean			±SD			Mean	±SD		*p*	
Serum vitamin D level, ng/mL		18.3			4.6			21.7	4.7		**< 0.001**	
Vitamin D Binding Protein, ng/mL		195.9			64.1			232	72.2		**< 0.001**	
		Vitamin. D level before treatment ng/mL			Vitamin. D level after treatment ng/mL			VDBP levels before treatment (ng/mL)			VDBP level after treatment (ng/mL)	
		Mean	±SD	*P*	Mean	±SD	*P*	Mean	SD	*P*	Mean	SD
Nutritional status	Normal weight	18.1	9.3	0.992	21.6	7.0	193.3	63.2	63.2	0.979	278.4	89.9
	Overweight	18.1	1.2		21.2	2.5	189.6	43.6	43.6		233.5	47.8
	Obese	18.3	4.0		20.7	4.5	196.5	62.9	62.9		229.7	70.9
Menopausal status	Premenopausal	20.1	4.7	**0.011**	22.8	5.6	224.3	72.7	72.7	**0.029**	257.6	82.6
	Postmenopausal	17.4	3.6		19.8	3.9		57.0	57.0		210.5	70.6

*SD* standard deviation; paired sample t-test was used for comparison between 2 groups; *p* < 0.05 was considered significant, ANOVA was used for comparison between more than 2 groups. Bold values indicate statistical significance (*p* < 0.05)

### Association of serum vitamin D and VDBP levels before and after treatment with tumor features

Higher tumor grades and stages were significantly associated with lower vitamin D_3_ and VDBP levels before and after treatment (*p* < 0.001 for each) (Table 3).

**Table 3:** Association of vitamin D_3_ and VDBP levels before and after treatment with tumor features

		Vitamin D_3_ level before treatment, ng/mL			Vitamin D_3_ level after treatment, ng/mL			VDBP level before treatment, ng/mL		VDBP _level_ after treatment, ng/mL		
		Mean	±SD	*P*	Mean	±SD	*p*	Mean	±SD	*P*	Mean	±SD
Side	Left	18.0	4.2	0.727	20.3	4.7	0.537	187.5	62.0	0.447	220.4	72.2
	Right	18.4	4.1		21.0	4.7		201.3	63.9		239.6	72.3
Stage	CIS	27.5	3.2	**< 0.001**	32.5	2.3	< 0.001	356.5	6.3	**< 0.001**	432.7	7.3
	I	25.7	2.4		28.6	3.3		295.1	27.5		340.7	42.2
	II	18.6	0.6		20.6	1.9		204.0	50.6		230.7	52.8
	III	15.5	2.9		18.1	3.5		153.7	51.8		193.4	61.7
Pathological types	IDC	18.1	3.9	0.480	20.6	4.6	0.561	194.7	64.4	0.441	229.9	72.8
	ILC	21.3	4.7		23.1	5.6		226.1	58.7		258.9	70.2
	DCIS	17.6	2.4		21.3	2.8		217.2	46.7		270.7	24.6
	Mixed	19.1	-		23.3	-		259.4	-		298.8	-
	Others	16.6	5.1		17.9	5.1		143.7	45.5		182.4	53.4
Grades	I	25.5	2.8	**< 0.001**	28.9	3.6	**< 0.001**	303.4	36.1	**< 0.001**	351.9	56.3
	II	18.0	0.8		20.3	1.6		192.8	47.4		227.9	53.0
	III	12.5	2.8		14.8	3.3		109.6	36.8		138.6	44.2

*SD* standard deviation; independent sample t test was used for comparison between 2 groups, ANOVA was used for comparison between more than 2 groups; *p*<0.05 was considered significant. Bold values indicate statistical significance (*p* < 0.05)

### Association of serum vitamin D3 and VDBP levels with tumor biomarkers and hormone receptor variables

The presence of triple negative molecular type and Ki67 above 20% was significantly associated with lower vitamin D_3_ and VDBP levels before and after treatment, while ER, PR, and Her2 did not significantly differ according to vitamin D3 and VDBP levels before and after treatment (*p* > 0.05 for each) as illustrated in Table 4.

**Table 4:** Association of vitamin D3 and VDBP level before and after treatment with biomarkers and hormone receptor status variables

		Vitamin D_3_ level before treatment, ng/mL			Vitamin D3 level after treatment, ng/mL			VDBP level before treatment, ng/mL		VDBP _level_ after treatment, ng/mL			
		Mean	±SD	*P*	Mean	±SD	*p*	Mean	±SD	*P*	Mean	±SD	*p*
ER	Negative	16.4	4.6	0.061	19.4	5.8	0.220	163.5	46.2	0.057	205.7	63.5	
	Positive	18.8	3.0		21.1	3.5		204.5	61.3		239.0	71.2	
PR	Negative	17.3	5.0	0.201	20.3	6.0	0.635	183.1	60.0	0.336	221.6	67.0	
	Positive	18.7	2.9		20.9	3.3		201.6	59.4		236.7	69.0	
Her2	Negative	18.0	4.4	0.458	20.3	4.7	0.359	187.3	56.6	0.201	225.5	67.4	
	Positive	18.7	3.7		21.4	4.6		210.7	68.5		243.3	72.5	
Triple negative	Absent	19.3	**3.3**	**< 0.001**	21.3	4.3	**0.006**	205.7	68.7	**0.005**	240.7	78.7	
	Present	11.4	**2.5**		17.0	5.2		135.8	41.0		179.1	67.2	
Ki67	Low	20.4	3.7	**< 0.001**	22.9	4.6	**<0.001**	230.0	65.9	**< 0.001**	266.2	77.2	
	High	15.9	3.1		18.4	3.6		158.7	54.7		194.8	61.3	

*SD* standard deviation; independent sample t test was used for comparison between 2 groups, *p* < 0.05 was considered significant. Bold values indicate statistical significance

(*p* < 0.05)

### Association of serum vitamin D3 and VDBP levels with pathological response to treatment

pCR was significantly associated with higher vitamin D3 and VDBP levels before and after treatment (*p* < 0.001 for both) that is shown in Table 5.

**Table 5:** Association of vitamin D_3_ levels by HPLC and VDBP by ELISA before and after treatment with response to treatment

Response	Serum Vitamin D levels before treatment ng/mL			Serum Vitamin D levels after treatment ng/mL			VDBP level before treatment, ng/mL			VDBP level after treatment, ng/mL	
	Mean	±SD	*P*	Mean	±SD	*p*	Mean	±SD	*P*	Mean	±SD
Complete Response	19.9	3.5	**< 0.001**	22.8	4.0	**<0.001**	225.1	62.3	**< 0.001**	265.7	71.8
Partial Response	16.6	2.0		18.1	1.3		159.3	51.1		181.1	55.9
No Response	9.7	1.9		11.6	2.2		59.9	16.7		108.4	11.1

*SD* standard deviation; ANOVA was used for comparison between more than 2 groups; *p* < 0.05 was considered significant. Bold values indicate statistical significance (*p* < 0.05)

### Correlation of serum vitamin D3 and VDBP levels with different clinical and laboratory variables

**Fig. 2:**
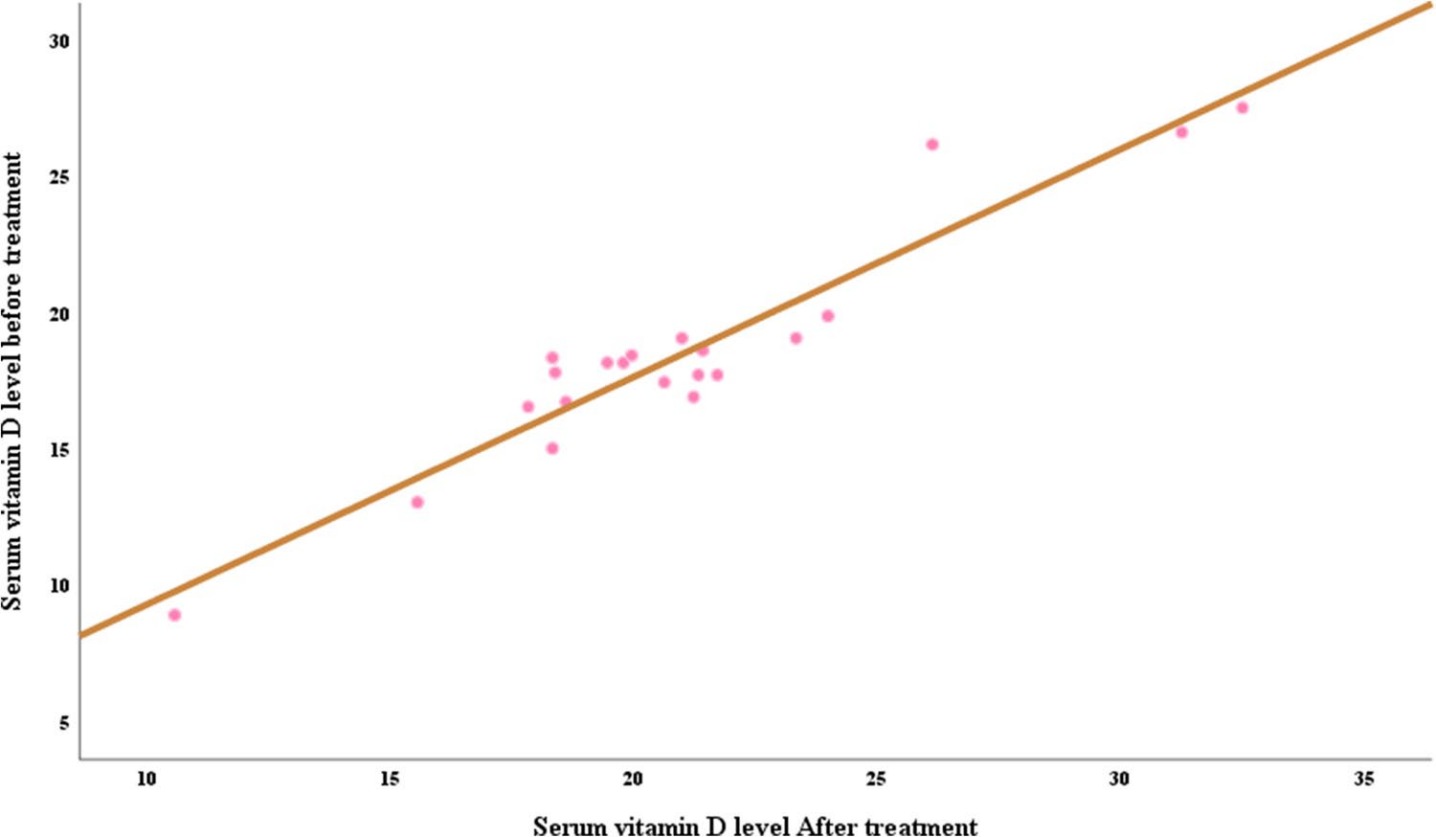
Correlations of vitamin D before and after treatment in BC cases

**Fig. 3:**
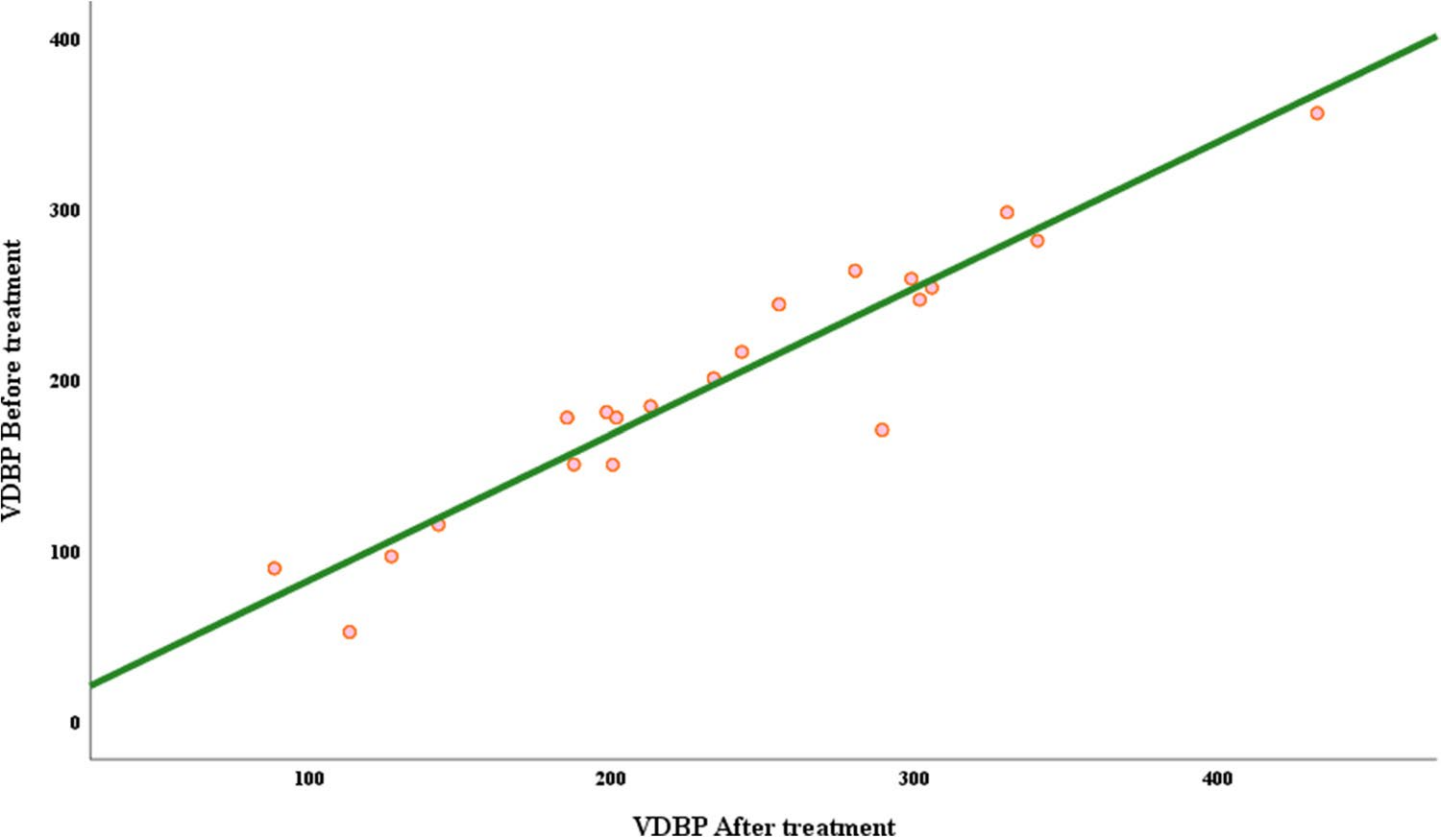
Correlations of VDBP before and after treatment in BC cases

Vitamin D3 level before and after treatment showed significant negative correlation with tumor stage and grade and significant positive correlation with VDBP (*p* < 0.001). VDBP level before and after treatment showed significant negative correlation with BMI, tumor stage, and grade and showed significant positive correlation with vitamin D3 (Table 6 and Figs. 2 and 3).

**Table 6:** Correlations of vitamin D_3_ and VDBP levels before and after treatment with age, BMI, stage, grade, CA 15-3

	Vitamin D_3_ level before treatment		Vitamin D_3_ level after treatment		VDBP level before treatment, ng/mL		VDBP level after treatment, ng/mL
	*r*	*P*	*r*	*p*	*r*	*P*	*r*
Age	–0.126	0.128	–0.180	0.133	-0.211	0.077	–0.143
BMI	–0.073	0.546	–0.191	0.111	-0.281	**0.043**	–0.306
Stage	–0.914	**< 0.001**	–0.591	**< 0.001**	-0.633	**< 0 .001**	–0.549
Grade	–0.824	**< 0.001**	–0.809	**< 0.001**	-0.703	**< 0.001**	–0.685
CA 15–3	–0.125	0.299	–0.146	0.225	-0.064	0.595	–0.054
VDBP	0.758	**< 0.001**	0.842	**<0.001**	0.758	**<0.001**	0.842

*r* Pearson's correlation coefficient. Bold values indicate statistical significance (*p* < 0.05)

### Prediction of pathologic response of BC patients to NACT

Significant predictors by univariable analysis were CA 15-3, triple negative molecular type, Ki67, baseline vitamin D, and baseline VDBP, while by multivariable analysis, the only predictors were triple negative molecular type and baseline level of vitamin D (Table 7).

**Table 7:** Prediction of BC response to NACT

	Uni variable				Multivariable		
	*p*	OR	95% CI		*p*	OR	95% CI
Age	0.187	1.016	0.993	1.039			
BMI	0.693	0.989	0.939	1.043			
menopausal status	0.242	1.470	0.771	2.801			
Comorbidity	0.814	0.934	0.526	1.656			
CA 15-3	**0.047**	1.012	1.000	1.024	0.058	1.027	0.999
Triple negative	**< 0.001**	3.720	2.739	5.051	**0.015**	1.488	1.109
Ki 67	**0.007**	2.278	1.249	4.154	0.099	1.166	0.188
Baseline vitamin D	**< 0.001**	0.522	0.378	0.722	**0.002**	0.506	0.331
Baseline VDBP	**< 0.001**	0.983	0.977	0.990	0.113	0.992	0.981

*OR* odds ratio, *CI* confidence interval. Logistic regression test was used. Bold values indicate statistical significance (*p* < 0.05)

## Discussion

Despite advances in systemic therapy, variability in treatment response continues to pose a significant clinical challenge. Identifying modifiable biologic factors that may influence therapeutic efficacy is therefore a priority. Recent data have linked low vitamin D levels to a wide range of diseases, including cancer, cardiovascular disease, autoimmune disease, and infection. 1,25(OH)2D, also known as calcitriol, is the biologically active form of vitamin D and exerts its action by binding to an intracellular receptor (VDR). VDR, first identified in a breast cancer cell line in 1979, belongs to the superfamily of nuclear receptors for steroid hormones and regulates gene expression by acting as a ligand-activated transcription factor. In addition to its main function of maintaining extracellular calcium levels, the activation of VDR influences up to 200 genes that mediate cellular growth, differentiation, and apoptosis. A number of factors influence the photosynthesis and bioavailability of vitamin D and contribute to the risk of impaired vitamin D status. These factors include variation in sun exposure, season, time of day, atmospheric components, clothing, sunscreen use, and skin pigmentation, as well as age, obesity, and the incidence of several chronic illnesses (Tsiaras and Weinstock 2011; Shao et al. 2012).

This is a prospective observational study including 71 BC patients The study was conducted in a university hospital serving poor farmers in the Nile Delta who are sharing to a great extent similar sun exposure, diets, clothing, and skin color. A statistically significant increase in both vitamin D3 and VDBP levels was observed following NACT (*p* < 0.001). Lower pre- and post-treatment levels of vitamin D3 and VDBP were significantly associated with postmenopausal status, higher tumor grade and stage, triple-negative molecular subtype, and high Ki-67 expression (*p* < 0.001). Conversely, higher levels of vitamin D3 and VDBP were significantly associated with achieving pCR (*p* < 0.001). Both vitamin D3 and VDBP levels demonstrated a significant negative correlation with tumor stage and grade (*p *< 0.001). Multivariable analysis showed that only triple-negative molecular subtype and the baseline vitamin D3 levels were independent predictors of pCR (OR = 1.488, 0.506, respectively) (95% CI = 2.73–5.05 and 0.37–0.72, respectively). Although these findings suggest a potential predictive role for vitamin D3 and VDBP in pathologic response, it is important to stress that causal relationships cannot be established due to the study design.

Hu et al. (2018) published their meta-analysis concerning the effect of serum vitamin D level on overall breast cancer survival. Six studies with a total number of 5984 patients were included. A highly significant linear dose–response relationship between circulating 25-OH-D levels and overall survival in patients with BC was reported.

Similar to our present results concerning vitamin D and breast cancer, other studies reached the same conclusion for bladder cancer and hepatocellular carcinoma. Hashem et al. (Hashem et al. 2022) reported that serum 25-Hydroxyvitamin D was significantly decreased in bladder cancer patients, especially those with invasive tumor muscle group. Moreover, Ebrahim (Ebrahim et al. 2018) concluded that vitamin D enhanced the antitumor activity of 5-FU in hepatocellular carcinoma.

Recently, the meta-analysis of Ottaiano et al. (2024) covered data from 722 patients regarding NACT response and 1033 patients for progression-free survival. The results revealed a 22% reduction in the likelihood of non-response to NACT associated with adequate VD levels. Omodei et al. (2025), a randomized clinical trial was conducted with 80 women aged ≥45 years with BC who were eligible for NACT. Women were randomized into two groups: vitamin D group and daily supplementation with 2000 IU of cholecalciferol (*n* = 40) or placebo (*n* = 40), for 6 months. The primary outcome measure was the pCR rate. Women with BC undergoing NACT who received supplementation with 2000 IU of vitamin D were more likely to achieve a pathological complete response than women in the placebo group.

However, the mechanisms underlying these associations remain incompletely understood. Vitamin D3 has been shown to influence cell proliferation, apoptosis, angiogenesis, and immune modulation, processes that are highly relevant to tumor progression and response to therapy. It may also influence the tumor microenvironment, particularly through regulation of inflammatory pathways and immune cell infiltration. VDBP, beyond its role in vitamin D transport, exhibits anti-inflammatory and antioxidant properties and may contribute to immune surveillance via its conversion into macrophage-activating factor (DBP-MAF) (Delrue and Speeckaert 2023). These mechanistic hypotheses merit further investigation in preclinical and interventional models.

Our neoadjuvant treatment did not include targeted or immunotherapy as these medications were not available for institutional neoadjuvant policies by the time the study started. It is worth noting that the addition of trastuzumab to neoadjuvant chemotherapy roughly doubles the proportion of patients with HER2-positive breast cancer who achieve pCR. Patients with pCR show better prognoses compared with those with residual disease after neoadjuvant therapy. Moreover, neoadjuvant immune checkpoint inhibitors improve efficacy outcomes in early-stage triple-negative and PD-L1+ hormone receptor positive/HER2- tumors with an acceptable safety profile (Takada and Toi 2020; Villacampa et al. 2024 Oct 1).

Methodological limitations of this study also need to be clarified. The sample size (*n* = 71) was relatively small, limiting statistical power and generalizability. Moreover, the cross-sectional and observational design limits the ability to infer directionality or causation. These limitations suggest that our findings should be viewed as hypothesis-generating. The results should not be interpreted as justification for routine vitamin D supplementation for improving chemotherapy outcomes without randomized controlled evidence.

Future directions should include larger randomized, multicenter cohorts with deeper exploration of the mechanistic role of VDBP and vitamin D3 in modulating tumor biology.

## Conclusion

Vitamin D3 and VDBP levels increased significantly after NACT and were positively associated with favorable clinical and pathological characteristics, including achievement of pCR. Triple-negative status and baseline vitamin D3 levels emerged as independent predictors of pCR. While these findings suggest a potential role for vitamin D3 in BC prognosis, ensuring adequate vitamin D_3_ levels—particularly in women at risk of deficiency—may be beneficial and warrants further investigation through controlled studies.

## Data Availability

The data sets generated and/or analyzed during the current study are available from the corresponding author on reasonable request.
